# TRAJECTORIES OF PAID AND FAMILY CARE TO SUPPORT ADULTS WITH DISABILITIES BY RURALITY

**DOI:** 10.1093/geroni/igae098.1694

**Published:** 2024-12-31

**Authors:** Katherine Miller, Yang Yang, Jennifer Reckrey, Katherine Ornstein, Jennifer Wolff

**Affiliations:** Johns Hopkins University, Baltimore, Maryland, United States; Johns Hopkins University, Baltimore, Maryland, United States; Icahn School of Medicine at Mount Sinai, Icahn School of Medicine at Mount Sinai, New York, United States; Johns Hopkins University, Baltimore, Maryland, United States; Johns Hopkins University, Baltimore, Maryland, United States

## Abstract

Family and friends are the dominant source of home-based care for older adults with disabilities (i.e., family caregivers). However, a growing share of older adults are receiving paid care (i.e., from a direct care worker such as a personal care aide). Differences in how paid and family care intersect by rurality are not well understood in the years immediately prior to death. We describe trajectories of paid and family care use in the five years prior to death using group-based multiple trajectory models stratified by rurality. We use a sample of community-dwelling National Health and Aging Trends Study respondents living with functional or cognitive limitations (n=2,549, rural n=481, urban n=2068). We examine the share of total hours of care provided by family versus paid caregivers. In both rural and urban cohorts, we find a 2-group model best fit our data: (a) “consistently high family care” (75% of the sample) and (b) “primarily paid care”. In both groups, the share of family caregiving decreases in concert with growth in paid care, but this shift was more pronounced among urban than rural residents (-5.2% vs. -3.6%). In the five years prior to death, rural residents have greater reliance on family caregivers relative to their urban peers. Notably, our findings that both rural and urban decedents rely primarily on family for care highlight the critical need to support family caregivers throughout the end-of-life trajectory.

